# Tracheostomy practice overview in cardiothoracic intensive care unit

**DOI:** 10.1186/2197-425X-3-S1-A743

**Published:** 2015-10-01

**Authors:** O Jansoniene, J Jones, T Tsoutsouras, D Hall

**Affiliations:** Royal Brompton and Harefield NHS Foundation Trust, Intensive Care, Harefield, United Kingdom; Royal Brompton and Harefield NHS Foundation Trust, Harefield, United Kingdom

## Introduction

The safety of tracheostomy practise in general intensive care units is widely recognised. The data about safety of tracheostomies after major cardiothoracic surgery or in high bleeding risk patients is less available.

## Objectives

The purpose of our study was to assess current practice of performing tracheostomies in patients after major cardiothoracic surgery, critically ill cardiology patients and patients with extracorporeal cardiac support, to evaluate the safety of procedure and determine the timing of tracheostomies and the duration of mechanical ventilation.

## Methods

We retrospectively collected the data of all patients in our cardiothoracic intensive care unit who underwent tracheostomy between October 2013 and September 2014. Total of 124 tracheostomies were performed in critically ill patients, out of which 79,2% were percutaneous and 20,8% surgical.

## Results

The median time from tracheal intubation to tracheostomy was 6 days (interquartile range 2-9 d). Thoracic patients received tracheostomies much earlier - median on day one of mechanical ventilation (interquartile range 0-3 d) compared to other patients (p = 0.002), they also had higher rate of surgical tracheostomies - 45,5%. For all the patients the median time to wean from mechanical ventilation after tracheotomy was 12 days (interquartile range 8-24 d), without any significant difference between the time and the type of procedure performed. Overall reported complication rate was 11,6% in percutaneous technique and 20% in surgical technique patients. Major complications occurred in 4 patients: in three cases percutaneous technique had to be converted to surgical approach due to failure, one patient had tracheal injury which was successfully managed conservatively. Most common complication was bleeding (occurred in 4.5% cases) but none of which required intervention or red blood cell transfusion. Bleeding incidence didn't differ in patients with or without being therapeutically anticoagulated nor being on double anti platelet treatment.

## Conclusions

The percutaneous tracheostomy is a preferred technique in our critical care unit. The median time from tracheal intubation to tracheostomy was 6 days. Percutaneous tracheostomy is a safe technique even in high bleeding risk patients with systemic anticoagulation and anti platelet therapy.

